# Binary Enterotoxin Producing *Clostridium perfringens* Isolated in Blood Cultures: Case Report and Review of the Literature

**DOI:** 10.3390/microorganisms12061095

**Published:** 2024-05-28

**Authors:** Linda Ben Saïd, Laure Diancourt, Audrey Rabeau, Virginie Gallet, Gauthier Delvallez, Marion Grare

**Affiliations:** 1 Department of Microbiology, Toulouse University Hospital, 31059 Toulouse, France; 2 National Reference Center for Anaerobic Bacteria and Botulism, Institut Pasteur, Université Paris Cité, 75015 Paris, Francegauthier.delvallez@pasteur.fr (G.D.); 3 Department of Thoracic Oncology, Toulouse University Hospital, 31000 Toulouse, France

**Keywords:** *Clostridium perfringens*, binary enterotoxin, BEC, blood culture, human infection

## Abstract

*Clostridium perfringens* (*C. perfringens*) is an anaerobic, spore-forming Gram-positive rod responsible for necrotizing gangrene, bacteremia in patients with cancer or gastrointestinal tract infection. *C. perfringens* virulence is due in large part to toxin production. In 2014, a new enterotoxin, BEC (binary enterotoxin of *Clostridium perfringens*) encoded by *becA* and *becB* genes, distinct from enterotoxin (CPE) encoded by the *cpe* gene, has been described. BEC-producing strains can be causative agents of acute gastroenteritis in humans. We present herein the case of a 64-year-old man who presented to the emergency department of Toulouse University Hospital with pneumonia and septic shock, without digestive symptoms. Blood cultures showed *C. perfringens* bacteremia and despite appropriate antibiotic treatment the patient passed away 7 h after admission. The characterization of the strain by whole genome sequencing revealed the presence of typical genes of *C. perfringens*: *plc* gene (alpha-toxin, phospholipase C) and *pfoA* (theta-toxin, perfringolysine). Surprisingly, this strain also harbored *becA* and *becB* genes encoding the recently described BEC toxin. Interestingly, alpha-toxin typing of our isolate and other published BEC isolates showed that they belonged to different PLC subtypes, confirming the high genetic diversity of these strains. To our knowledge, it is the first clinical case reporting bacteremia due to a BEC-producing *C. perfringens* isolate.

## 1. Introduction

*Clostridium perfringens* (*C. perfringens*) is an anaerobic, spore-forming Gram-positive rod and is a ubiquitous environmental bacteria, found in decaying vegetation, soil and feces, and is a normal constituent of the intestinal flora of humans and animals. *C. perfringens* is also a major pathogen of humans and livestock [1]. In humans, this bacterium is responsible for necrotizing gangrene, bacteremia in cancer patients or in patients with gastrointestinal tract infection, antibiotic-associated diarrhea, and infection after chemoembolization. Gas gangrene is the most frequent manifestation of *C. perfringens* infection and is one of the fatal manifestations that present after trauma or gastrointestinal surgeries [2,3,4,5,6,7].

*C. perfringens* virulence is due in large part to toxins production. The six main virulence factors in pathogeny are alpha-toxin, beta-toxin, epsilon-toxin, iota-toxin, enterotoxin and NetB toxin, respectively, produced by *plc*, *cpb*, *etx*, *iap* and *ibp*, *cpe* and *netB* genes. Recently, the toxin-based typing scheme has been revised into seven toxinotypes (A–G) according to the presence or absence of these six virulence factors [8]. There is a link between toxin production patterns and clinical presentation in both human and animal infections. In human diseases, toxinotype A is responsible for classical gas gangrene, type C for enterititis necroticans, and type F for food poisoning, antibiotic-associated diarrhea, and sporadic diarrhea [1]. In 2014, a new enterotoxin, BEC (binary enterotoxin of *C. perfringens*), encoded by *becA* and *becB* genes, distinct from *C. perfringens* enterotoxin (CPE) encoded by the *plc* gene, was described. BEC-producing *C. perfringens* strains can be causative agents of acute gastroenteritis in humans [9].

We describe here the first case of bacteremia due to a BEC-producing *C. perfringens* isolate, without digestive symptoms and a fatal issue.

## 2. Case Presentation

A 64-year-old man presented to the emergency department of Toulouse University Hospital (Toulouse, France) with chest pain and dyspnea for a few days. At the admission, the patient was hemodynamically unstable (desaturation (65%), hypotension (84/46), tachycardia (120/min), febrile (39 °C)) and the physical examination reported marbling of the lower limbs, cold extremities and clinical signs of pneumonia (rhonchi, sign of respiratory distress). The patient did not present any gastrointestinal symptoms (corticotherapy may have masked digestive signs). A CT scan could not be performed due to the patient’s hemodynamic instability. Four days before, the patient received his last cure of chemotherapy (cycle two day 8 of paclitaxel, fourth line of treatment) for his epidermoid carcinoma PDL1 extended to the pleura diagnosed in December 2021. The first line of treatment consisted of four cures of carboplatin/paclitaxel/pembrolizumab, followed by three cycles of pembrolizumab only in April 2022. The clinical and radiological progression of the carcinoma directed physicians to introduce the second line of treatment with gemcitabine alone, and since November 2022, the third line with vinorelbinalone. In his medical history, this patient was also treated with dexamethasone once a week and pomalidomide 15/28 days for POEMS syndrome diagnosed in 2007 (three bone marrow transplants), and had steroid-induced diabetes. Since August 2022, he has been hospitalized two times for pneumonia (first without documentation, second in December 2022, with *Enterobacter cloacae* complex). Initial laboratory results revealed mild anemia (9.9 g/dL), thrombocytopenia (70 G/L), leucopenia (2.74 G/L) elevated troponin-I (29 ng/dL) and NT-proBNP (1224 pg/mL), and elevated C-reactive protein (CRP) (122 mg/L). Renal and hepatic parameters were without particularity. Blood cultures (peripherical and on PAC) were performed at admission and sent to the bacteriological laboratory. Antimicrobial treatment using piperacillin–tazobactam and amikacin were rapidly introduced. The patient had rapid clinical deterioration with the development of respiratory failure, shock and multiorgan failure. The patient passed away 7 h after admission.

Concerning the microbiological data, blood cultures were introduced less than one hour after collection in the BD FX Bactec (BD Diagnostics, Franklin Lakes, NJ, USA), and blood cultures growth in less than 5 h after collection, exclusively in anaerobic bottles. Microscopic examination with Gram staining showed large Gram-positive bacilli, with punctuated coloration. Twenty-four hours after inoculation on Columbia agar with 5% sheep blood (BD Diagnostics) and incubation in anaerobic conditions (anaerobe gas generation bag, Anaerogen Compact, Thermo Scientific, Waltham, MA, USA) at 35 °C, gray-white colonies with a double zone of hemolysis were observed. Mass spectrometry (Brucker Daltonics, Billerica, MA, USA) identified the isolate as *C. perfringens* with a good score. Antibiotic susceptibility testing (AST) was performed in accordance with the European Committee on Antimicrobial Susceptibility Testing (EUCAST) 2022 guidelines [10] and showed susceptibility to all tested antibiotics: amoxicillin, amoxicillin–clavulanate, piperacillin–tazobactam, clindamycin, vancomycin, rifampicin and metronidazole. The isolate was sent to the National Reference Center (NRC) for Anaerobic Bacteria and Botulism (Institut Pasteur, Paris, France) to confirm the identification and explore the toxin’s profile. The identification of *C. perfringens* was confirmed by a polymerase chain reaction (PCR) assay targeting the *plc* gene, encoding for alpha toxin [11]. In addition, the isolate tested positive for other genes: *pfoA* gene encoding for theta toxin [12], and *becA* and *becB* genes, encoding for the recently described BEC [9].

Genome sequencing was therefore performed. Briefly, genomic DNA extraction was performed on a 24 h bacterial liquid culture using the DNA Easy UltraClean microbial kit (QIAGEN, Courtaboeuf, France). Short-read genome sequencing was performed by the P2M Sequencing Platform at the Institut Pasteur (Paris, France) using the Illumina Nextera XT DNA Library Prep Kit and the NextSeq 500 system sequencer. Paired-end (PE) sequencing reads were clipped and trimmed with AlienTrimmer v. 2.0 [13] corrected with Musket v. 1.1 and subjected to a digital normalization procedure with ROCK v. 1.9.3 software. All programs were used with their default settings. The remaining processed reads were assembled and scaffolded with SPAdes v. 3.15.2 [14]. In silico detection of antimicrobial resistance (AMR) and toxin genes was performed with BioNumerics (v. 7.6.2, Applied Maths NV, Sint-Martens-Latem, Belgium). The assembled genome was also scanned against the *Clostridium perfringens* PubMLST typing scheme as described in Xiao et al. [15].

It confirmed that the isolate did carry *plc*, *pfoA*, *becA* and *becB* genes. Further, the genomic sequence showed that the isolate (2023/00056) did not carry any resistance genes, except *tetA(P)* and *tet(B)P*, encoding for a membrane tetracycline efflux protein. The multilocus sequence typing (MLST) approach showed that the isolate belonged to ST-754. Furthermore, based on the alpha-toxin (PLC) sequence [16], seven available *becAB* isolates were typed (Supplementary data, Table S1) and showed that the strain 2023/00056 belonged to a new PLC subtype. The isolate harbored at least two plasmids: one plasmid encoding the *tetA(P)* and *tet(B)P* genes, and one harboring *becA* and *becB* genes. The nucleotide sequence of *becA/becB* was identical to those reported, for all available sequences, except strain CP653-17 (98% identity).

To the best of our knowledge, we describe here the first case of bacteremia due to a BEC-producing *C. perfringens* isolate harboring *becA* and *becB* genes encoding for BEC, without digestive symptoms and a fatal issue.

## 3. Discussion

The *Clostridium* species is the second most common cause of anaerobic bacteremia after the *Bacteroides* species (0.5 to 2% of bacteremia), with *Clostridium perfringens* being the most frequently isolated species [3,5,17,18]. In population studies, the incidence rate varies from 1.8/100,000 person–years to 4.8/100,000 person–years [19,20], which makes *C. perfringens* bacteremia a rare pathology. At the Toulouse University Hospital microbiology laboratory, only 95 *C. perfringens* bacteremia cases have been reported for the 2010–2023 period (with a median of six cases per year), and 56% were monomicrobial.

The clinical relevance of *Clostridium* sp. bacteremia is debated in the literature. In an old paper, *C. perfringens* was considered clinically relevant only in 33% of cases, whereas for other *Clostridium* species, this rate was higher than 80% [21]. A recent paper showed opposite results with a high frequency of clinically relevant bacteremia, both for *C. perfringens* (76%) and other *Clostridium* species (91.2%) [18]. A recent study at the University Hospital of Montpellier (France) showed a link between the frequency of bacteremia implicating anaerobic bacteria (BIAB) and cancer patients [22]. BIAB frequency was higher in patients treated at a center specialized in cancer (ICM) than in those treated at Montpellier University Hospital (MUH) (10.4% vs. 4.9%, *p* < 0.01). In this study, *Bacteroides* and *Clostridium* were the most identified genera (64 and 18 episodes, respectively). They concluded that anaerobic bacteremia should especially be taken into account in cancer patients, which can be a high mortality risk factor [22]. As previously shown by other authors, *C. perfringens* bacteremia, although relatively uncommon, had a high 30-day mortality rate (near 30%, up to 43% in cancer patients) [3,5,18,19,20]. In our case, the causative role of *C. perfringens* bacteremia in patient death could not be established, despite the bacteria being isolated from three pairs of positive blood cultures. However, this patient presented a significant number of comorbidities (especially epidermoid cancer during the fourth line of chemotherapy) which could explain the impact of *C. perfringens* bacteremia.

Concerning risk factors for mortality, in a recent paper, Yamamoto et al. showed that all patients with *Clostridium* sp. bacteremia had malignancies (colorectal cancer, pancreatic cancer, gastric cancer…), and that the most common species isolated was *C. perfringens* followed by *C. ramosum*. Cancer and immunosuppression are consistently reported as the main comorbidities in patients infected with *C. perfringens* [3,5,19]. Age, cancer history and ineffective antibiotherapy were independently associated with increased hospital mortality [23]. In another study, age, absence of fever, high CRP level on arrival, high SOFA score, elevated lactate and the presence of sepsis or septic shock within 24 h from obtaining the first blood culture were all significantly associated with mortality [20]. Our patient had numerous comorbidities: epidermoid carcinoma PDL1 extended to the pleura with multiple chemotherapy failures, POEMS syndrome, and steroids-induced diabetes. At his admission, he presented a high CRP level and signs of septic shock.

Several studies underlined that early appropriate anti-anaerobe therapy, especially in patients with terminal cancer, contributes to improving patient survival in the case of anaerobe bacteremia [5,23,24]. *C. perfringens* is highly susceptible to beta-lactams and metronidazole, but resistance to clindamycin was common [20]. In this case, the genomic sequencing of the isolate showed the absence of resistance genes for the tested antibiotics, which is consistent with the phenotypic AST results. Empiric therapy of bacteremia and septic shock is often associated with beta-lactam, aminoglycosides and/or glycopeptides. Although antibiotic therapy is effective against anaerobes, and despite our patient receiving a piperacillin–tazobactam/amikacin treatment, which was appropriate, death occurred within a few hours.

Sequencing analysis by NRC revealed the presence of typical genes of *C. perfringens*: a *plc* gene encoding for alpha-toxin (phospholipase C) and a *pfoA* gene encoding for theta-toxin (perfringolysine). However, surprisingly, this strain also harbored *becA* and *becB* genes encoding the recently described BEC toxin. This toxin was previously described in four foodborne disease outbreaks in Japan [9,25], in a sporadic gastroenteritis case in Japan [26], in a healthy two-year-old child in the United Kingdom [27], and more recently in another sporadic case in Japan [16].

Interestingly, alpha-toxin typing of our isolate and other published BEC isolates showed that the isolates belonged to different PLC subtypes and confirmed the high genetic diversity of these strains [16,28].

Until recently, CPE was considered the only virulence factor responsible for the gastrointestinal symptoms reported in *C. perfringens* type F foodborne outbreaks. Like iota-toxin, BEC is a member of the actin-ADP ribosylating toxins family. It consists of two independent components, i.e., an enzymatic effector component (BECa) and a cell-binding component (BECb) [1]. In rabbits, it was shown that culture supernatants of BEC-positive strains caused fluid accumulation in intestinal loops [9].

Only a few publications describe this toxin and in a recent study including 585 *C. perfringens* human clinical isolates, genes encoding BEC were identified in only one strain [16]. So, the frequency of BEC-producing *C. perfringens* strains both in intestinal microbiota and in clinical specimens (blood cultures, digestive fluids, abscess…) remains unknown. Here we describe a case of BEC-producing *C. perfringens* bacteremia in a patient with many comorbidities, four days after his last cure of chemotherapy, and presenting clinical symptoms of pneumonia with septic shock signs. No digestive signs were reported. Fatal issues occurred seven hours after admission. *C. perfringens* positive blood cultures were the only collected specimen. However, no link could be established between bacteremia and death. The question arises of the involvement of this toxin, and the implication of other virulent factors, in this type of fatal case. As for *Clostridioides difficile*, asymptomatic carriers of toxigenic BEC *C. perfringens* isolates could be possible, as the pediatric case described by Kiu et al., and asymptomatic patients may constitute a potential reservoir, highlighting the clinical importance of surveillance and monitoring of BEC-producing *C. perfringens* isolates in the context of public health [27,29].

In conclusion, we report here, to the best of our knowledge, the first case of BEC-producing *C. perfringens* bacteremia described in a patient without digestive symptoms and with fatal issues. So, further research is required to understand the importance of BEC-producing *C. perfringens* both in human foodborne gastroenteritis and in other pathologies related to *C. perfringens*.

More data and studies are needed before a possible inclusion of BEC production in a future expansion of the toxinotype scheme of *C. perfringens*.

## Data Availability

The whole genome sequence of the isolate 2023/00056 (BioProject number PRJEB65712) was deposited in the European Nucleotide Archive’s reference sequence database under accession number CAUJRZ010000000 and is available at https://www.ebi.ac.uk/ena/browser/view/CAUJRZ010000000, accessed on 18 June 2023. The Sequence Read Archive accession number was ERS16308088.

## Supplementary Materials

The following supporting information can be downloaded at: https://www.mdpi.com/article/10.3390/microorganisms12061095/s1, Table S1: Alpha-toxin (PLC) types of seven BEC *C. perfringens* isolates.
