# Supplementary material for: Binary Enterotoxin Producing Clostridium perfringens Isolated in Blood Cultures: Case Report and Review of the Literature
Source: Microorganisms. 2024 May 28;12(6):1095. doi: 10.3390/microorganisms12061095 (PMC11205559; doi:10.3390/microorganisms12061095)
Supplement: Supplementary file 1 [file microorganisms-12-01095-s001.zip › microorganisms-2965109-supplementary.pdf]

Table S1: Alpha-toxin (PLC) types of seven BEC *C. perfringens* isolates

		Key amino acids [*a]																					
Isolate [Accession number]	Alpha-toxin (PLC) type	8	**13**	22	40	47	**54**	57	71	166	176	195	202	205	209	222	244	277	362	363	365	**373**	375
CP482.2020 [MW665543]	If	A	**A**	A	A	V	**L**	N	E	A	H	A	**A**	**T**	K	R	Y	P	**I**	**S**	A	**I**	A
A18.256 [AP024979]	IIa	A	**A**	A	A	V	**L**	N	E	A	H	A	D	A	K	R	D	P	F	P	A	**V**	A
TS1 [AP024973]	IIa	A	**A**	A	A	V	**L**	N	E	A	H	A	D	A	K	R	D	P	F	P	A	**V**	A
OS1 [AP024969]	IId	A	**A**	A	A	V	**L**	N	E	A	H	**V**	D	A	K	R	D	P	F	P	A	**V**	A
O13-19 [AP024976]	IVb	A	**T**	A	A	**I**	**L**	N	E	A	H	A	D	A	K	R	D	P	F	P	A	**V**	A
CP653 [MH900557]	IVb	A	**T**	A	A	**I**	**L**	N	E	A	H	A	D	A	K	R	D	P	F	P	A	**V**	A
2023/00053 [CAUJRZ010000000]	IV-new	**V**	**T**	A	A	V	**L**	N	E	A	H	**V**	D	A	K	R	D	P	F	**S**	A	**V**	A

[*a] : Key amino acids previously described in Aung et al, 2021: positions 13, 54 et 373 in red were used for the alpha-toxin typing (I-IV); positions in blue defined subtypes.
